# Reward context determines risky choice in pigeons and humans

**DOI:** 10.1098/rsbl.2014.0451

**Published:** 2014-08

**Authors:** Elliot A. Ludvig, Christopher R. Madan, Jeffrey M. Pisklak, Marcia L. Spetch

**Affiliations:** 1 Department of Psychology, University of Warwick, H1.21 Humanities Building, Coventry, UK; 2 Department of Psychology, University of Alberta, Edmonton, Canada

**Keywords:** risk sensitivity, decision making, gambling, comparative cognition

## Abstract

Whereas humans are risk averse for monetary gains, other animals can be risk seeking for food rewards, especially when faced with variable delays or under significant deprivation. A key difference between these findings is that humans are often explicitly told about the risky options, whereas non-human animals must learn about them from their own experience. We tested pigeons (*Columba livia*) and humans in formally identical choice tasks where all outcomes were learned from experience. Both species were more risk seeking for larger rewards than for smaller ones. The data suggest that the largest and smallest rewards experienced are overweighted in risky choice. This observed bias towards extreme outcomes represents a key step towards a consilience of these two disparate literatures, identifying common features that drive risky choice across phyla.

## 1. Introduction

Humans and other animals often display different patterns of risk preferences. Humans are generally risk averse when faced with a choice between safe and risky gains [1]. Non-human animals ranging from insects to primates also sometimes exhibit risk aversion for amounts of food reward [2,3] but are instead risk seeking when faced with variable delays [4] or negative energy budgets ([5], but see [6]). Moreover, chimpanzees (*Pan troglodytes*) [7,8], rhesus monkeys (*Macaca mulatta*) [9,10] and pigeons (*Columba livia*) [11–13] can even be risk seeking for amounts of food reward. Here, we attempt to reconcile these conflicting findings by testing pigeons and humans in formally identical procedures.

One prominent difference in how risky choice is assessed in humans and non-human animals is the manner in which information is conveyed [14]. Whereas humans are typically presented with described odds, animals, by necessity, are tested through repeated experience with the rewards. This experience-based choice more closely resembles the natural environments of animals and the ancestral environment for humans. Recent research has revealed that this information format influences human risky choice and can even reverse risk preferences [15–17].

For example, Hayden & Platt [9] tested humans in a task designed to closely follow risky-choice experiments in monkeys [18]. Instead of the typical one-shot described choices [1], they gave humans multiple trials to learn the outcomes. Under these conditions, humans did not show consistent risk aversion, but rather showed a win–stay, lose–shift strategy similar to monkeys [9,18].

**Figure 1.**
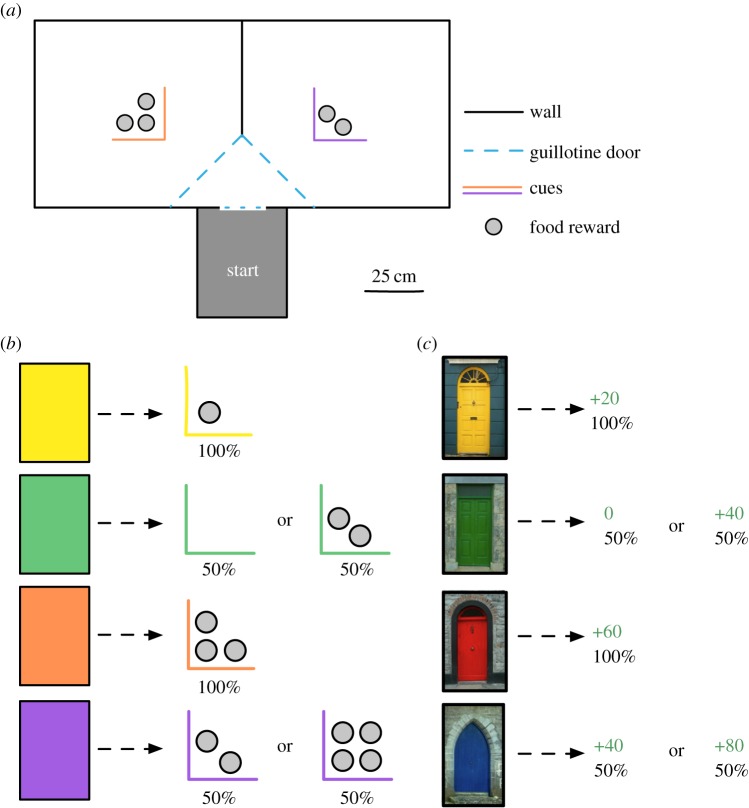
Task schematics. (*a*) Testing arena for pigeons. Pigeons would enter from the start box and choose which half of the arena to enter through the guillotine doors. (*b*) Stimuli and reward contingencies for pigeons and (*c*) for humans. For both species, the cues led to either a low–safe reward, low–risky reward, high–safe reward or high–risky reward (from top to bottom). (Online version in colour.)

In this paper, we took the opposite strategy and transformed an experimental protocol used for evaluating learned decisions in humans [16,17,19] into a pigeon foraging analogue (see figure 1). Individuals of both species learned about four options: two that led to *high-value* rewards and two that led to *low-value* rewards. For each reward level (high or low), one *safe* option yielded a guaranteed fixed reward, and one *risky* option yielded a 50/50 chance of a better or worse reward. The expected values for the safe and risky options were matched within each reward level.

The risk-sensitive foraging literature suggests that risk sensitivity is determined by the coefficient of variation (CV = standard deviation/mean) of the reward amount [2,3]. In our experiment, variance was fixed across reward levels, making the CV smaller for the decisions with high-value rewards. Therefore, individuals should show lower risk sensitivity for these high-value decisions than for the low-value decisions. By contrast, prior behavioural results with humans suggest that the extreme outcomes (highest and lowest rewards) are overweighted in decisions from experience [19,20]. As a result, more risk seeking should be observed in high-value decisions than low-value decisions.

Thus, both the risk-sensitive foraging literature and the human risky-choice literature lead us to predict an effect of reward value on risky choice, but the foraging literature predicts an effect on risk sensitivity, whereas the human literature predicts an effect on risk preference. In either case, our key prediction is that using choice protocols where all consequences are learned from experience should lead to similar choice patterns across species.

## 2. Material and method

### (a) Subjects

Six pigeons (*C. livia*) were housed individually and kept at 85% of ad libitum weight through post-session feeding. Twenty-seven human participants (*Homo sapiens*: 21 females, age (*M* ± s.d.) = 19.2 ± 1.1 years) were recruited from the University of Alberta participant pool, and informed consent was obtained.

### (b) Procedure

Figure 1*a* illustrates the layout of the test arena used for pigeons. On each trial, pigeons exited a start box and were allowed to observe the coloured cues on each side, which concealed the rewards (food cups) on that trial. After the pigeon made its choice by entering one side of the arena, a guillotine door closed behind them, and they were allowed to gather the reward for that trial. Figure 1*b* details the reward contingencies. The four different-coloured cues indicated both reward level (high or low) and risk level (safe or risky). The low-value safe option yielded one food cup; the low-value risky option yielded either zero or two cups with a 50/50 chance. The high-value safe option yielded three food cups; the high-value risky option yielded either two or four cups with a 50/50 chance.

Figure 1*c* illustrates the task used for humans, which consisted of clicking on coloured doors on a computer screen. As with pigeons, there were four options (doors). The low-value safe door always yielded 20 points, and the low-value risky door yielded either 0 or 40 points with a 50/50 chance. The high-value safe door always yielded 60 points, and the high-value risky door yielded either 40 or 80 points with a 50/50 chance. Doors usually appeared in pairs on the screen, and after each selection, feedback for the selected door was provided on the screen for 1.2 s. Humans were requested to maximize points.

Both species received three kinds of trials: on *decision trials*, participants chose between the safe and risky options with equal expected values (high or low). On *catch trials*, participants chose between options of unequal expected value (high versus low). On *forced-choice trials*, only one option was available and needed to be selected, ensuring that participants experienced all contingencies.

Pigeons were tested in daily sessions of 10 trials and received at least 72 instances of each decision trial. Humans received six blocks of 48 trials in a single session, including 72 instances of each decision trial.

## 3. Results

**Figure 2.**
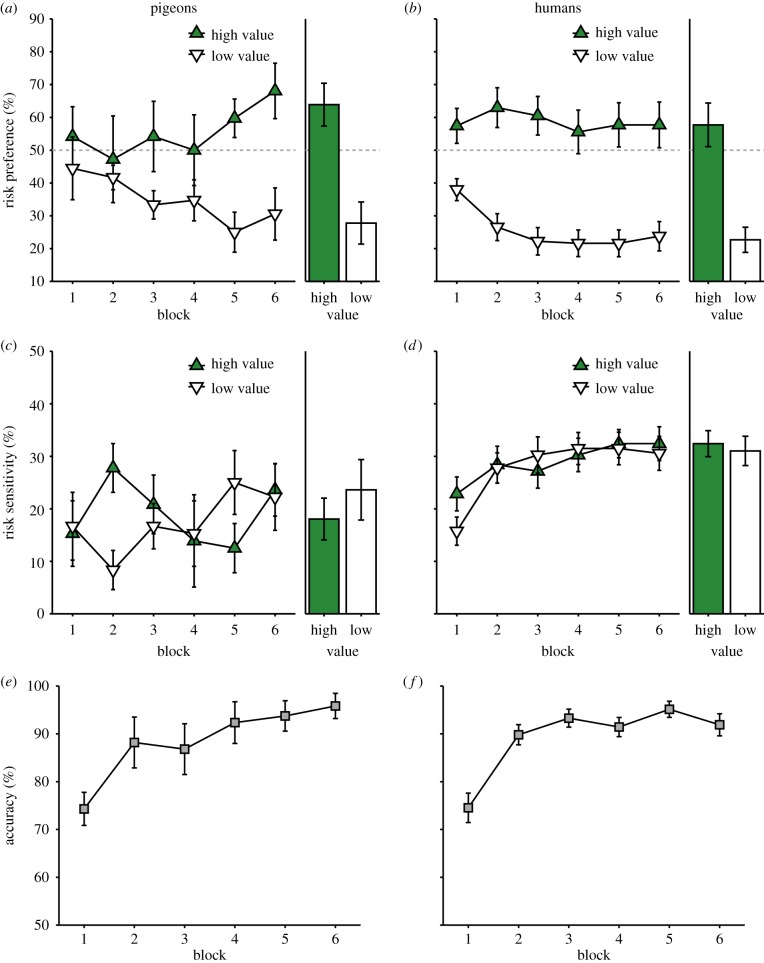
Behavioural results. (*a*) Risk preference (%) for pigeons and (*b*) for humans and (*c*) risk sensitivity (%) for pigeons and (*d*) for humans over blocks of 12 trials. Bar plots (right) show average performance over the final two blocks. (*e*) Response accuracy (%) on catch trials over blocks of 12 trials for pigeons and (*f*) for humans. (Online version in colour.)

Both pigeons and humans were less risk averse for the high-value options than the low-value options, even trending towards outright risk seeking by the end of training. Figure 2*a*,*b* plots the risk preference across training and shows that, by the end of training, both species exhibited less risk aversion to the high-value cues. Risk sensitivity was operationalized as the absolute value of the deviation in risk preference from risk neutrality (50% [3]). Figure 2*c*,*d* shows that risk sensitivity did not consistently differ for high-value and low-value cues. Figure 2*e*,*f* shows that both species readily learned the task.

Consistent with extreme outcomes driving choice, both species were more risk seeking for high-value decisions than for low-value decisions. Collapsed across the final two blocks (right panel of 2*a*), pigeons chose the risky option for the high-value option about 35% of the time more often than for the low-value option (*t*_5_ = 4.91; *p* = 0.004, Cohen's *d* = 2.50). Similarly (right panel of 2*b*), humans also chose the risky option about 35% of the time more often for the high-value than for the low-value option (*t*_26_ = 4.88; *p* < 0.001, *d* = 1.27). By contrast, neither species showed significant differences in risk sensitivity between the high- and low-value decisions as predicted by the CV hypothesis [pigeons: *t*_5_ = 0.43, *p* = 0.69, *d* = 0.35; humans: *t*_26_ = 0.56. *p* = 0.58, *d* = 0.16].

## 4. Discussion

Pigeons and humans showed remarkably similar patterns of risky choice, with both species showing risk aversion for low-value rewards and a tendency towards risk seeking for high-value rewards. This convergence suggests a phylogenetic generality of the behavioural mechanisms underlying risky choice. The results are consistent with the potential overweighting of the biggest and smallest rewards in the context in choice [19,21]. In this case, overweighting the biggest loss (0 points or no food) in low-value decisions produces risk aversion, and overweighting the biggest gain (80 points or four food cups) in high-value decisions produces more risk seeking. This overweighting could be implemented through many different theoretical mechanisms, including the distortion of an internal utility function [1] or changes in the samples used for relative comparisons [22]. These results do not support the CV hypothesis [2,3], which predicts lower risk sensitivity for the high-value decisions than the low-value decisions. There were no reliable differences in risk sensitivity between high- and low-value decisions for either species.

These results corroborate and extend previous studies showing that risky choice in humans was influenced by the most extreme rewards in the decision context. Notably, choice between any particular safe and risky option depended on the other choices in the context. If the risky option led to the best possible outcome, people were more risk seeking and if it led to the worst outcome in the context, people were risk averse [19]. The risk preferences also correlated with a bias towards better remembering the most extreme outcomes [21]. The results presented here suggest that, similar to humans, pigeons also overweight the most extreme outcomes in a decision context.

Several alternative explanations for these results can also be ruled out. For both the high- and low-value decisions, the energy budget was equivalent as both decisions occurred during the same session and followed large or small rewards equally often. Thus, a change in local energy budget cannot explain these results. Alternatively, the risk aversion observed in the low-value case could possibly be ascribed to a strong aversion to the zero reward, rather than an aversion to the extreme value. This aversion, however, does not explain the trend towards risk seeking observed with the high-value rewards. Moreover, in previous work with humans, we found the same pattern of risky choice when all reward values were shifted so that there was no zero reward [14].

These results build on a growing literature, showing that, despite superficial differences, risky choice in humans and other animals can be made similar [9,12]. The key factor appears to be use of experience-based choice procedures for humans that match those typically used in animal studies [14]. Here, through such a well-matched procedure, we identified a shared behavioural process—the overweighting of extreme outcomes—which drives risky choice with experienced rewards.

## 

Procedures were approved by University of Alberta ethics committees.

## Data accessibility

Additional details are available in the electronic supplementary material.

## Funding statement

This study was financially supported by the Alberta Gambling Research Institute (AGRI) and Natural Sciences and Engineering Research Council (NSERC) of Canada.

## Supplementary Material

Supplementary Methods
