# Supplementary material for: Reward context determines risky choice in pigeons and humans
Source: Biol Lett. 2014 Aug;10(8):20140451. doi: 10.1098/rsbl.2014.0451 (PMC4155912; doi:10.1098/rsbl.2014.0451)
Supplement: Supplementary Methods [file rsbl20140451supp1.docx]

**Supplementary Methods**

**1. Pigeon Experiment**

(a) Subjects

The subjects consisted of six pigeons (*Columba livia*, 3 Silver King and 3 Racing pigeons), all with previous experience in learning experiments. All six pigeons were kept on a 12-hour light-dark cycle with light onset at 0600. The pigeons were housed individually within metal cages, fed a diet of commercial pigeon pellets (Mazuri Ex Gamebird Breeder Diet, PMI International), and had their food intake adjusted daily following the experimental sessions to maintain 85% of their free-feeding body weight. A seventh Silver King bird started training, but failed to learn the task altogether, exhibiting a strong side bias, and was not included in the experiment. Both grit and water containing a dissolved vitamin supplement were left freely available to the birds outside of the experiment.

(b) Apparatus and Stimuli

Fig 1A provides a schematic of the testing arena. The arena consisted of two 90x82-cm compartments separated by a 50-cm long central wall, surrounded by a white curtain. The central and rear walls were white-painted plywood, and the front and side walls were made of white plastic. Two white plastic 43×63-cm guillotine doors, each set at a 45° angle from the central wall, were positioned so as to form a small triangular *decision area* against the front of the arena. Both doors were operated via a single-string pulley system. Birds entered the decision area through a 16×20-cm entrance from a start box. For half the birds, the start box was 22×40 cm and white, and for the remaining birds, the start box was 48×39 cm with black walls and a white ceiling and a removable barrier blocking entry into the decision area. A thin layer of aspen chip bedding was laid down prior to the experiment in the testing arena.

Choices were cued visually by four different-colored stimuli (purple, yellow, green, and orange). The stimuli consisted of two horizontal 23×60-cm planks of 2.5-cm thick plywood set at a 90° angle, covered with laminated paper of the appropriate colour. Food rewards were presented in 6.8-cm diameter cups, located behind each choice stimulus. Each cup always contained three pigeon pellets on top of a small amount of grit. A ceiling-mounted closed-circuit camera was used to monitor behaviour from an adjacent room.

(c) Procedure

*Pretraining.* Birds were trained on the task in three phases. First, all pigeons were hand shaped to enter each compartment from the start box through the guillotine door, walk to the furthest corner to obtain three food cups, and then return to the start box. Once the pigeons reliably completed 10 trials in this manner, a white (neutral) choice stimulus was added to the centre of each compartment. The pigeons were gradually shaped to walk around this stimulus to obtain the cups, which were now obscured by the stimulus, and return to the start-box as before. Once they could reliably complete 10 trials in this manner, pigeons received 64 forced-choice trials, 16 with each of the 4 coloured cues, randomly distributed over the course of seven experimental sessions. At the start of each trial, one guillotine door opened allowing entry into that side. When the pigeon entered the arena, the door was closed, and the pigeon was allowed to collect the rewards available. Following reward consumption, the guillotine door was re-opened allowing the pigeon to return to the start-box, which now contained a single food reward.

The four coloured cues had different reward contingencies, varying in reward value (low or high) and risk (safe or risky). For the *low-value* cues, the safe option always had 1 food cup behind it, and the risky option had 0 or 2 cups behind it with a 50/50 chance. Each food cup contained three food pellets. For the *high-value* cues, the safe option always had 3 food cups behind it, and the risky option had 2 or 4 cups behind it with a 50/50 chance. Presentation of the stimuli was randomly counterbalanced across the 64 trials such that each cue was presented equally often on both sides. Colour was randomly counterbalanced across pigeons.

*Testing.* Test sessions consisted of 10 trials each and contained 2 forced-choice trials (as above) and 8 choice trials. The 8 choice trials consisted of 4 *decision trials* and 4 *catch trials*. On decisions trials, the pigeon chose between the two stimuli that led to the risky and safe outcomes with equal expected values (low or high). On catch trials, the pigeon chose between two stimuli that led to outcomes with unequal expected values (high vs. low), and served to assure that the pigeons were learning the task. Catch trials always pitted the two safe options or the two risky options against one another. Sessions were counterbalanced so that each stimulus appeared twice on each side. Trial order was randomized. The testing procedure was identical to the end of pretraining with the exception that both guillotine doors opened simultaneously. On forced-choice trials, one of the compartments was now empty. If the pigeon happened to enter the empty side, both guillotine doors were left open until the pigeon entered the side with the stimulus. Testing lasted 40 sessions, and all pigeons received at least 72 of both types of decision trials. There was no formal inter-trial interval; each trial followed immediately after the end of the previous trial once the apparatus was reset (which took on average about 30 s).

**2. Human Experiment**

(a) Participants

Twenty-seven introductory psychology students at the University of Alberta participated for course credit. One additional participant was excluded for not learning the contingencies (see below). Each participant gave written informed consent.

(b) Procedure

Participants played a computer-based task, and they were instructed to try to earn as many points as possible. Participants were recruited and received instructions in groups, but performed the task on computers located in individual rooms. Figure 1C illustrates the task. On each trial, participants saw pictures of 1 or 2 doors on a computer screen and selected one by clicking on it. Choices were immediately followed by feedback for 1.2 s, which displayed the points won along with a cartoon graphic of a pot of gold. Feedback was only given for the chosen door. Total accumulated points were continuously displayed on the screen. An inter-trial interval of 1 to 2 s separated trials.

Sessions consisted of 6 blocks of 48 trials. Each block included a mixture of trial types: There were 24 *decision trials* that required a choice between either the 2 low-value doors or 2 high-value doors (12 of each). On *high-value* decisions, the safe door led to 60 points, and the risky door led equiprobably to 40 or 80 points. On *low-value* decisions, the safe door led to 20 points, and the risky door led equiprobably to 0 or 40 points. There were 16 *catch trials* that required a choice between a high-value door and a low-value door (4 for each combination of safe and risky door). These trials allowed us to assess whether participants were engaged in the task and learned the contingencies. Data from 1 participant who chose the high-value door on fewer than 60% of these catch trials was excluded. On 8 *forced-choice trials*, there was only one door, which had to be selected to continue. Each door appeared exactly twice per block on these trials, which guaranteed that all reward contingencies were experienced, even if some doors were initially unlucky, and participants tended not to select them. Trial order was randomized within blocks. Each door appeared equally often on either side of the screen and in combination with all the other doors. Door color was counterbalanced across participants. Blocks were separated by a short riddle to provide a brief break and limit inattentive responding.

Data available from the Dryad Digital Repository: <http://doi.org/10.5061/dryad.NNNNN>.

**Acknowledgments**

The authors would like to thank Roberta Lee, Ariel Greiner, Nuha Mahdi, and Josh Young for help with running the experiments and Isaac Lank and Al Denington for help in building the apparatus.
